# Book Reviews

**Published:** 1892-04

**Authors:** 


### Book Reviews.

A Practical Treatise on the Diseases of Women. By T. Gaillard Thomas, M. D., LL. D., Emeritus Professor of Diseases of Women in the College of Physicians and Surgeons, New York, and Paul F. Mundé, M. D., Professor of Gynecology in the New York Polyclinic. New (sixth) edition, thoroughly revised and rewritten by Dr. Mundé. In one large and handsome octavo volume of 824 pages, with 347 illustrations. Cloth, $5.00; leather, $6.00. Philadelphia: Lea Brothers & Co. 1891.

It is twenty-four years since Thomas first appeared as an author upon the subject of Diseases of Women. For twelve years, or one-half of that period, he held undisputed sway in America, and was quoted as the supremest authority upon all subjects pertaining to operative as well as minor gynecology. Not only this, but the marked favor with which the early editions of his work was received in Europe is evidenced by the fact that it was translated into German, French, Italian, and Spanish, and, hence, obtained a widespread circulation throughout continental Europe. During this period five editions of the work were published,—an average of one edition for every two years and a half of its life.

From the fifth edition, which appeared in 1880, down to the one now under consideration, no attempt has been made to revise or keep the work abreast of the progress of gynecological science. This may seem strange, for it is really during this period of quiescence on the part of Thomas as an author that gynecology has made its greatest strides, overleaping all the barriers which seemed to lie athwart its path, and trampling down all the traditions which the early teachers had built upon. It must be confessed that much of the teaching of the early editions has become obsolete, or nearly so, hence the volume has very much depreciated as a guide to students or practitioners. To let such a valuable treatise die during the life of the author, seemed to be almost inexplicable, and we can well understand how the pride of Thomas in his earlier editions, the reception they received both in America and Europe, and his popularity as a teacher, would conspire to prevent, if possible, such a misfortune to his book. The difficulty seems to have been to find a person who would revise the author's work to his satisfaction, and at the same time bring it forward to conform, in main respects, to the requirements of the present day.

The selection of Dr. Paul F. Mundé was in every point of view a happy solution of the difficulty. Dr. Mundé ranks as one of the progressive gynecologists of the period, and is at the same time sufficiently conservative on many subjects to bring him into sympathy with Thomas, and the men of his age and trend of thought. We have examined the sixth edition of Thomas, so thoroughly revised by Mundé, with some degree of care, and we are of the opinion that it would be difficult to find more faithful work on the part of a reviser than the pages of this book exhibit. Wherever there has been a difference of opinion between the author and the editor, the latter has not failed to make himself distinctly understood upon such points of difference. These notes of the editor are included in brackets, and serve to add to the interest of the work.

To show how complete a change of front often takes place in the mind of an author, with reference to some questions as years progress, let us cite an instance. In Thomas's first edition, and for the succeeding four issues, he was an advocate of sponge tents, as well as those made of other material, for the dilatation of the os uteri under certain conditions. On page 98 we find the following in brackets:

NOTE.—Since the last edition, Dr. Thomas's position in regard to tents has changed to such a degree that in a communication on the subject received from him after this chapter had gone to press, he says: "Tents of all kinds should be discarded in gynecology, absolutely and completely. Dilatation of the os uteri can be more certainly and completely accomplished by rapid dilatation by the divulsor under anesthesia and careful antisepsis, and should always be practised by preference." I am not personally prepared to go as far as this, and should be sorry to be compelled to part with the tupelo tent in certain cases where a gradual, slow, and thorough dilatation is desired. Sponge and laminaria I have not employed for years. Feeling that a sweeping condemnation of tents in gynecology would scarcely meet with the approval of the profession, and believing, as I do, in their limited utility, I have thought it my duty to endeavor to instruct the practitioner how to use them with as little risk as possible, and have, therefore, retained this section.

In this position we lean towards the opinion of the author as against that of the editor. We are perfectly willing to admit that a tupelo tent in the hands of Dr. Mundé, or in the hands of men with experience equal to his, may be a safe instrument, but we are of the further opinion that it is safer to adhere to the position taken by the author, in a text-book at least, than to that of the editor. We believe that gynecology can afford to part with all kinds of tents, and if it should it will not thereby be the loser.

Dr. Mundé has introduced a short chapter on Electricity as a Therapeutical Agent in Gynecology. Since there are a number of well-meaning physicians who think they have discovered in electricity the agent which is the *summum bonum* of therapeusis, it is well to have this subject dealt with in a text-book, in an intelligent fashion, by a man who has a considerable experience with it, and one who is willing to record its failures as well as its presumed successes. Such a man is Dr. Mundé, and the profession is familiar with his opinions in reference to this agent, since they have been published from time to time in the *American Journal of Obstetrics*. But we are of the opinion that Dr. Mundé's electrical friends will feel that he has done the subject up in too summary manner in the seven pages that he devotes to it in this book. For ourselves, we are quite content that he has taken up no more valuable space with an agent that is little understood, and, we fear, has in it less possibilities for good than for harm in the hands of the average practitioner. Only the exceptionally few experts can be trusted not to do damage with it, and even they are not sure as to how much cure there is in it.

A new chapter has also been introduced on Hermaphrodism, one on Diseases of the Urethra and Bladder, and one on Diseases of the Female Breast. The chapters on Parauterine Cellulitis, and on Pelvic Peritonitis, have been revised with the intention of bringing them down to the latest date, but we are sorry to find omitted some of the principles that have been recently taught by Tait, Price, McMurtry, and others, on this interesting and important subject. The chapter on Pelvic Hematocele also might bear some further reconstruction in view of its intimate relationship to Extrauterine Pregnancy. The strong points in Thomas's book are where he deals with disorders of menstruation with the use of pessaries and with plastic operations on the genital tract.

Taken all in all, it is a most valuable guide to the student and practitioner, and is a work which the gynecologist must not exclude from his book shelves. We cannot commend too highly the faithful work that Dr. Mundé has performed in revising this book, and his labors entitle him to the gratitude of his readers everywhere. A large number of new illustrations have been introduced, and the press-work is of the best.

---

Wood's Medical and Surgical Monographs, consisting of Original Treatises and Reproductions, in English, of Books and Monographs selected from the latest literature of foreign countries, with all illustrations, etc. Published monthly. Vol. XII. No. 1. October, 1891. No. 2, November, 1891, and No. 3, December, 1891. Price, $10.00 a year; single copies, $1.00. New York: William Wood & Co. 1891.

The articles in the October number are: Treatment of Diseases of Women, by Thure Brandt; The Modern Treatment of the Morphine Habit, by Dr. A. Fromine; A Contribution to the Study of So-called Scarlatina Puerperalis, by Prof. Dr. Renvers; The Influences of Alcohol upon the Organism of the Child.—A Pharmacological-Clinical Study, by Prof. R. Demme; The Diseases of Development, by Dr. J. Comby. The first paper is of sufficient importance to demand a critical examination.

About a hundred years ago, Mr. Ling, the father of modern gymnastics, and the greatest gymnast of his time, then professor at the Military Academy of Carlberg, Stockholm, proposed and taught what he called sick-gymnastics, his idea being that nearly all diseases of the race might be ameliorated or cured by proper movements and manipulations of the different parts of the body, if intelligently applied. The idea thus communicated to his pupils has never been lost sight of in Sweden, though its value remained obscured for many years. Within the last three or four decades the subject has received the attention of the medical world, and many intelligent physicians have recognized its therapeutic value, chiefly under the form known as massage, and the theory of the treatment seems to rest on a scientific basis. While this appears to be admitted by good professional authority, we may question its adaptability to general gynecological practice, so much depending on a certain mechanical aptness, almost amounting to sleight-of-hand, which seems to be possessed by the author, and which may be difficult to transmit to others, however well fitted they may be otherwise for the work. Perhaps we can give no more succinct idea of the author's method than by quoting a passage from one of the opening paragraphs.

He says :

> In the Summer of 1847, a patient called upon me for relief from a suddenly developed prolapse of the rectum. As medical aid could not be obtained at once, and I was ignorant of the medical treatment of such cases, I conceived an idea, which was carried out in the following manner : I placed the patient in a bent semi-recumbent position, pushed the right hand in the left hypogastrium deep into the pelvis, and made traction inward and upward, combined with gentle shaking. In this way I proposed to obtain hold below the flexure of the large intestine and thus draw the gut mechanically upwards. This succeeded so well that the gut was reduced and has not again prolapsed.

Though this will give an idea of Mr. Brandt's method, nothing but a careful perusal of the monograph will give any idea of its successful application to nearly every disease to which the viscera of the female pelvis are subject.

Copious illustrations of the various gymnastic movements are given in rather rude but intelligent wood-cuts, and schools for teaching its intelligent use have been established in various parts of the world. In the treatise before us, however, we have the experience of a gentleman who has a wide European reputation as a successful manipulator in the direct application of his art to many diseases heretofore supposed to be beyond any aid but the surgeon's knife. Mr. Brandt is not a physician, though it is evident that he is well acquainted with the anatomy and physiology of the human frame, especially of the pelvic organs, which have formed the principal parts to which he has devoted his attention.

One word by way of caution: While we do not question the propriety or usefulness of Mr. Brandt's mechanical treatment of pelvic diseases when their serious nature may require more or less heroic practice, we are satisfied that abuse may attend an indiscriminate use of massage, as tending to unnatural excitement of the nervous system, more especially in organs of great sensibility. Great as is the latitude of the physician in professional treatment of physical ailments, he ought to remember that his honor is pledged that no needless act of his wounds the natural modesty of his patient.

In the November number are found the following: The Practice of Hypnotic Suggestion, being an Elementary Hand-book for the Use of the Medical Profession, by George C. Kingsbury, M. A., M. D.; A Practical Manual of the Bacteriological Analysis of Water, by Dr. Miquel.

The December number contains: Modern Materia Medica, with Therapeutic Notes, by Dr. Otto Roth; Index to Vol. XII. This number closes the series, and their publication ceases. We are sorry to lose these valuable periodicals, for they have furnished a vast amount of excellent literature at a most reasonable cost. We advise those who have not already obtained the entire series to send their orders to the publishers at once, before they are out of print.

---

A Practical Treatise on Diseases of the Ear, including a Sketch of Aural Anatomy and Physiology. By D. B. St. John Roosa, M. D., LL. D., Professor of the Diseases of the Eye and Ear in the New York Post Graduate Medical School, and President of the Faculty; Surgeon to the Manhattan Eye and Ear Hospital; Consulting Surgeon to the Brooklyn Eye and Ear Hospital; formerly Professor of Ophthalmology in the University of the City of New York, and of Diseases of the Eye and Ear in the University of Vermont; formerly President of the Medical Society of the State of New York, etc., etc. Seventh revised edition. New York: William Wood & Co. 1891.

Otology has finally become recognized the world over as a distinct specialty. It is no longer considered as a matter of course that an ophthalmologist must also treat the ear, though frequently men are found who cultivate both specialties. The eye and ear have no special anatomical relationship, and whoever would practise either or both of these specialties must necessarily study them as distinct entities, only controlled and linked together by that same general law which makes any one organ of the body an essential factor to the complete and perfect whole. Dr. Roosa has been for twenty years as recognized authority in diseases of the ear, which is the line upon which he has won his greatest renown, though he is well known also in the department of ophthalmology. When the first edition of this book appeared, now almost nineteen years ago, the distinguished author had already an experience of nearly four thousand cases upon which to base his observations and from which to deduce his conclusions. He has revised and reissued the book from time to time, keeping it abreast of the progress of otology in every respect, and now, after nearly twenty years from its first appearance, it is presented to us with the ripened judgment of a master.

The historical sketch of the progress of otology, which occupies the first forty pages of the book, is one of the most interesting sketches of the march of science in a special department of medicine that we have ever read, and it will repay any student or practitioner to study it carefully as a medical classic. Dr. Roosa's style is so clear and forcible that he cannot be misunderstood when he lays down a proposition, a law, or when he teaches a fact. In view of the recent prevalence of influenza, and the marked secondary conditions that it leaves along its trail, involving the upper air passages, and, in many instances, the internal ear with them, it would be well for the general practitioner to study carefully such a treatise as Roosa has given us, that he may have some understanding of the nature of the structures that have been invaded, and be able to place an intelligent interpretation upon the symptoms that lurk in the wake of "la grippe." More even than this, let every student obtain Roosa's book and make it his guide in his after professional life, so far as he may have to do with the ear, either directly or indirectly, as a specialist or general practitioner. Roosa's book must be to American medicine an authority upon the ear, as Gruber's is to the German school of medical science.

The book is well illustrated, handsomely printed, and copiously indexed, and commends itself to professional favor even now stronger than ever, notwithstanding the fact that so many rivals have sprung up since it first appeared. The author is still a young man, with all the vim and enthusiasm of youth, coupled with the strength and maturity of middle age. We expect that Roosa will be quoted as authority in this specialty of otology for at least twenty years to come. He is a safe teacher and a brilliant practitioner in this somewhat subtle, though interesting and important, department of medical science.

---

A SYSTEM OF PRACTICAL THERAPEUTICS. Edited by HOBART AMORY HARE, M. D., Professor of Therapeutics and Materia Medica in the Jefferson Medical College, Philadelphia. Assisted by WALTER CHRYSTIE, M. D., late Physician to St. Clement's Hospital, and Instructor in Physical Diagnosis in the University of Pennsylvania. Volume I., General Therapeutic Considerations, Prescription-Writing, Remedial Measures other than Drugs, Preventive Medicine, Diathetic Diseases, and Diseases of Nutrition. With illustrations. Volume II., Fevers, Diseases of the Respiratory System, Circulatory System, and Hematopoietic System, and Diseases of the Digestive System. With illustrations. Philadelphia: Lea Brothers & Co. 1892.

This work, as its title indicates, is of an encyclopedic nature, and there are seventeen contributors to Volume I. besides the editors. Volume I. deals with General Therapeutic Considerations, by Horatio C. Wood, M. D.; Prescription-writing and the Combination of Drugs, by Joseph P. Remington, Ph. D. These two titles may be considered as introductory. Then comes the section on Remedial Measures other than Drugs, and under this class we find Electro-Therapeutics, by A. D. Rockwell, M. D.; the Rest-Cure for Neurasthenia and Hysteria, by John K. Mitchell, M. D.; Swedish Movements and Massage, by Benj. Lee, M. D.; General Exercise, by Edward Mussey Hartwell, M. D.; Climate, by Edwin Solly, M. R. C. S.; and Hydrotherapy and Mineral Springs, by Simon Baruch, M. D. Then follows the section on Preventive Medicine, including General Sanitation, by Henry B. Baker, M. D.; Disinfection, by Geo. M. Sternberg, M. D.; Antisepsis and Asepsis, by J. William White, M. D.; Nutrition and Foods, including the Treatment of Obesity and Leanness, by I. Burney Yeo, M. D.; and, finally, Diathetic Diseases and Diseases of Nutrition are discussed under the following heads, viz.: Tuberculosis, by Solomon Solis-Cohen, M. D.; Scrofula and Rachitis, by Walter Chrystie, M. D.; Acute and Chronic Articular Rheumatism, Rheumatoid Arthritis, and Gout, by James Stewart, M. D.; Scurvy, by John B. Hamilton, M. D.; Diabetes Mellitus, by Frederick A. Packard, M. D. This volume consists of 1052 handsomely printed pages, and closes with an elaborate index.

Volume II. has twenty-six contributors, exclusive of the editors, and opens with an article on Syphilis, by Robert W. Taylor, M. D., which is a conclusion of the section of Diathetic Diseases and Diseases of Nutrition, commenced in Volume I. The next section is upon Fevers, and we find the first title Scarlet Fever, Measles, Rotheln, and Varicella, by J. Lewis Smith, M. D.; then follow in their order Small-Pox, by William M. Welch, M. D.; Typhoid Fever, by Frederick P. Henry, M. D.; Typhus Fever, by Manuel Dominguez, M. D.; Malarial Diseases and Dengue, by George Dock, M. D.; Yellow Fever, by Jerome Cochran, M. D.; Cerebro-Spinal Fever, by J. C. Wilson, M. D. The next section is on Diseases of the Respiratory System, and embraces Diseases of the Nasal Chambers, by Ralph W. Seiss, M. D.; Diseases of the Pharynx and Larynx, by Charles E. Sajous, M. D.; Diphtheria and True Croup, by J. Chalmers Cameron, M. D.; Asthma, Acute and Chronic Bronchitis, and Whooping-Cough, by James T. Whittaker, M. D.; Pulmonary Emphysema, Atelectasis, Abscess, and Gangrene, by M. Howard Russell, M. D.; Croupous and Catarrhal Pneumonia, by Edwin E. Graham, M. D.; Diseases of the Pleura, by Rudolph Matas, M. D.

The next section is on Diseases of the Circulatory System and Hematopoietic System, and includes Acute and Chronic Diseases of the Heart, by W. H. Thomson, M. D.; Nervous Diseases of the Heart, by T. Lauder Brunton, M. D.; Diseases of the Blood-vessels and Diseases of the Blood, by Frederick C. Shattuck, M. D.; Diseases of the Liver, Gall-bladder, Hepatic Ducts and Spleen, by J. H. Musser, M. D.; Diseases of the Thymus and Thyroid Glands, and Exophthalmic Goitre, by Richard C. Norris, M. D.

The final section of this volume is on Diseases of the Digestive System, and embraces Diseases of the Mouth and Salivary Glands, including Mumps, by A. D. Blackader, M. D.; Acute and Chronic Gastric Catarrh, Gastric Atrophy, Gastric Ulcer, Gastric Cancer, and Gastric Dilatation, by D. D. Stewart, M. D.; Cholera Morbus, Cholera, Cholera Infantum, and Dysentery, by Frederick E. Packard, M. D.; Obstruction of the Intestines, by Edward Martin, M. D.; Peritonitis, Appendicitis, and Perityphlitic Abscess, by Roswell Park, M. D.; and Diseases of the Rectum and Anus, by Charles B. Kelsey, M. D.

This volume contains 1158 pages, including a voluminous index, and is in every way the counterpart of Volume I., in its paper, press-work, and general make-up, which are all of the very best that experienced medical book-makers can produce. It is expected that Volume III., to be issued soon, will close this valuable contribution to medical and surgical therapeutics, which has been a stupendous undertaking for the young and accomplished editor.

---

A TEXT-BOOK OF PHYSIOLOGY. By M. FOSTER, M. D., LL. D., F. R. S., Professor of Physiology in the University of Cambridge, England. Fourth American, from the Fifth English edition, thoroughly revised. Octavo, 1072 pages, 282 engravings. Cloth, $4.50 ; leather, $5.50. Philadelphia : Lea Brothers & Co. 1891.

The author of this book is not a stranger to the professional world. His fame as a teacher is known on both sides of the Atlantic, and his text-book has been accepted as a standard authority on physiology in Europe and America. This fourth American edition has been enriched by some changes and additions, but the general character of the treatise is much the same as was presented in previous editions. The changes made are chiefly explanatory, and do not affect especially the underlying principles that prevail in the physiological laboratory. The author does not claim that this treatise is especially rich in histological research, though he has introduced many statements in histology that deserve to be considered and stated as a part of the physiological teaching, having kinship to the general subject of physiology. The English edition of the book appeared in parts, but these have been grouped in one volume by the American editor, whose name, however, does not appear. It is quite unnecessary to undertake a critical review of a standard treatise on physiology that has already passed through several editions, and has become accepted authority. We have examined the book before us with sufficient care to satisfy ourselves that Foster is an author who has the enthusiasm of the student, together with the balanced judgment and skill of the teacher. This last edition of his treatise will most easily find for itself a place on the book shelves of every teacher of physiology in our American schools, and must be given place in the list of text-books as a guide to the student in his laboratory work. An appendix has been added, giving the chemical basis of the animal body, which is an interesting addendum to a useful and elaborate treatise. The volume is produced by the publishers in a most excellent form, and deserves to rank among the handsomest issues from this famous publishing house.

INTERNATIONAL CLINICS: A Quarterly of Clinical Lectures on Medicine, Surgery, Gynecology, Pediatrics, Neurology, Dermatology, Laryngology, Ophthalmology, and Otology. By Professors and Lecturers in the leading Medical Colleges of the United States, Great Britain, and Canada. Edited by JOHN M. KEATING, M. D., Philadelphia, Consulting Physician for the Diseases of Women to St. Agnes' Hospital, Philadelphia; Editor Cyclopedia of Diseases of Children.---J. P. Crozer Griffith, M. D., Philadelphia, Clinical Professor of Diseases of Children in the University of Pennsylvania; Professor of Clinical Medicine in the Philadelphia Polyclinic.---J. Mitchell Bruce, M. D., F. R. C. P., London, England; Physician and Lecturer on Therapeutics at the Charing Cross Hospital; and David W. Finlay, M. D., F. R. C. P., London, England; Physician to the Middlesex Hospital, and to the Royal Hospital for the Diseases of the Chest; Lecturer on Clinical Medicine in the Middlesex Hospital Medical School. Large octavo. Pp. viii,---356. Illustrated. July, 1891. Philadelphia: J. B. Lippincott Company. 1891.

The second number of this series is even an improvement on the first, and it is full of interesting material, presented in a most readable form. Beginning with a biographical sketch of the late Dr. Joseph Leidy, accompanied with a full page portrait of this distinguished teacher and scientist, it goes on with its thirty-nine lectures by both American and foreign teachers, who are, for the most part, recognized authority on the subjects that they deal with. Among the titles we may mention the Pathology of Angina Pectoris, by Ernest Sansom, M. D., of London; Chronic Diffuse Nephritis, by James Tyson, M. D., of Philadelphia; The Treatment of Displacements and Flexions of the Uterus, by Egbert H. Grandin, M. D., of New York; Myoma, Complicating Pregnancy, etc., by Paul F. Mundé, M. D., of New York; Effects of Double Ovariotomy and Oophorectomy on the Secondary Sexual Characters of Women, by J. Bland Sutton, F. R. C. S., of London; Fracture of the Spine, Operation and Recovery, by M. Allen Starr, of New York; Peripheral Nervous System, etc., by James J. Putnam, of Boston; Acute Inflammation of the Middle Ear, by Charles H. Burnett, of Philadelphia. We have picked out at random some of the most important of these lectures, to give our readers an idea of their general scope and import; they are of value and of interest, and will well repay careful perusal. We consider this series of clinics a valuable addition to the literature of medicine, the publishers having put them out in excellent manner.

Medical Symbolism, in connection with Historical Studies in the Arts of Healing and Hygiene. Illustrated. By Thomas S. Sozinskey, M. D., Ph. D., author of The Culture of Beauty, etc. Physician's and Student's Ready Reference Series, No. 9. Pp. 171. Philadelphia and London: F. A. Davis, publisher. 1891.

To the practitioner who has no time or regard for matters not strictly practical, this little work would mean nothing; but to him who wishes to understand not only the essentials but also the non-essentials of medicine,—and these latter go far toward making a cultivated physician,—we can heartily commend this work. To an intelligent body of men, the history of medicine and medical mythology should be well known. How many of us know the meaning of the symbol written many times daily at the head of a prescription? The author says it is an "obsolete talisman."

The original form of it appears to have been a figure like a Z, with the lower horizontal part crossed with a scepter-shaped line. This, or a modification of it, has been from time immemorial the symbol of the planet Jupiter. Hence the reason for placing it at the head of prescriptions; for the great planet was believed in other days to have a favorable influence over diseases.

The medical symbols of recent times are fully explained: the physician's gold-headed cane, gold ring, the pentacle, etc., etc.,—all in all making a pleasant and instructive hour's reading.

Innumerable references are given to prove the author's assertions and deductions, and the result is an interesting and entertaining essay. W. C. K.

---

The Principles of Bacteriology: A Practical Manual for Students and Physicians. By A. C. Abbott, M. D., First Assistant, Laboratory of Hygiene, University of Pennsylvania. Philadelphia. With illustrations. Pp. 263. Philadelphia: Lea Brothers & Co. 1892.

The study of bacteriology must be a rapid-growing one, judging from the continual ripening of text-books on the subject. The discovery of new facts and new pathogenic organisms, no doubt, are in part accountable, because even this admirable little treatise, published in 1892, contains no account of the latest acquisition to our collection. This work is intended for laboratory students and "practitioners of medicine," but we doubt very much whether practitioners pay much attention to practical bacteriology. The scope of the work is excellent and very practical, serving as a guide for students at work in a laboratory. The description of the various methods, apparatus, media, and the different genera are careful and painstaking, which, aided by well-executed engravings, makes it a desirable little book to possess.

Lea Brothers & Co. have clothed it nicely, as is their custom.

W. C. K.

---

PUBLIC LEDGER ALMANAC FOR 1892. George W. Childs, Publisher. Philadelphia.

This convenient little book contains a complete calendar for 1892, and a record of the important events of the past year in Philadelphia; also a list of the churches and public buildings of that city. Among other valuable items of information is a list of prominent government officials at Washington, an army and navy record, and a record of the census. Very seldom has so much desirable information been condensed into so small a space, and the generosity of the publisher in furnishing such a useful book free of cost is unexampled.

